# Supplementary material for: Simulating the progression of the COVID-19 disease in Cameroon using SIR models
Source: PLoS One. 2020 Aug 25;15(8):e0237832. doi: 10.1371/journal.pone.0237832 (PMC7447022; doi:10.1371/journal.pone.0237832)
Supplement: Attachment — Submitted filename: Response to Reviewers.pdf [file pone.0237832.s001.pdf]

Review response letter

Dear Editor,

Thank you for giving us the opportunity to revise our paper on "Simulating the progression of the COVID-19 disease in Cameroon using SIR models" The suggestions offered by the reviewers have been helpful and we also appreciate your insightful comments on revising some aspects of the paper.

We have included the reviewers' comments immediately after this letter and responded to them individually, indicating how we addressed each concern and describing the changes we have made.

Best regards,

Ulrich NGUEMDJO
Freeman MENO
Audric DONGFACK
Bruno VENTELOU

Reviewer 1:

General comments:

1. A general revision of English language, syntax and grammar is necessary.

Response:

Thank you for the comment, we have used the services of a professional language editor

2. Footnotes must instead be incorporated into the text or properly added as references following the journal's citation style.

Response:

Done, thank you for the comment.

3. Figures should have captions in a style as dictated by the journal, with a more extensive legend.

Response:

Done, thank you for the comment.

4. Gallicisms, such as using the French spelling of the name of the Republic of Cameroon, should be corrected.

Response:

Done, thank you for the comment.

5. The Authors analyse data available until April 10th, and its association with public health interventions. Given that more than one month has passed after this date, the Authors can comment and provide more information on the current situation and the impact of the measures taken.

Response:

Thank you for the comment. We provided more information on the current situation and impact of the measures taken in the country in the discussion/conclusion of our manuscript (second and third paragraphs).

Specific comments:

1. "The rest of the study is organized…" - this detail the structure of the paper (which is unnecessary), not the study itself. That would be collecting data from the Cameroonian

ministry of health, applying the model, performing simulations for varying rates of exposure and transmittance and evaluating the data.

Response:

Thank you for this comment. We have modified the sentence to make it clearer.

2. "The political capital, Yaoundé"

Response:

Done, thank you for the comment.

3. Line 41/54: The headings "The Model" and "The SIR Model" are redundant with each other. The sections should be merged.

Response:

Thank for this comment, the two sections were merge.

4. Line 45: Provide a source for the sentence "This model..... a large population".

Response:

Thank for thing comment. We provided a source for the sentence.

5. Lines 45-46: When first explaining the model, names of the population fractions shouldn't be capitalized ("Susceptible", "Infectious", "Recovered"), instead the abbreviation should be put in brackets: "susceptible (S)", "infectious (I)", "recovered (R)".

Response:

Done, thank you for the comment.

6. Lines 47-48: "the last group was modified to "Removed"" - here a clearer explanation is needed, for example "was modified to "Removed" in order to cover/account for individuals who can no longer transmit the disease for reasons other than acquired immunity, such as being placed in quarantine, hospitalized, or dying".

Response:

Thanks for this comment. We have modified the sentence to make it clearer as suggested.

7. Line 56: "an exponentially distributed time" - Time is not distributed, but determined exponentially with a decay rate of γ.

Response:

Done, thank you for the comment.

8. Line 60: Why does "close contact" need to be in quotes? Also, it should be merged with the next sentence: "… an individual has close contact with the rest of the population over the remaining time at a rate determined by the parameter β (also known as effective contact rate), which is the product of the average number of exposures per unit of time (τ) and the likelihood of infection at each occasion of exposure (μ)." The equation ($\beta = \tau \times \mu$) should be included in its own paragraph below.

Response:

Thanks for this comment. We have modified the sentence to make it clearer as suggested.

9. Line 107: Consider substituting "if nothing is done indeed" by "if no action is taken".

Response:

Done, thank you for the comment.

10. Line 150-155: There lacks a clear, mathematical expression of the relationship between hygienic measures and the probability of infection upon contact. It needs to be framed in terms of the removal rate and its connection to flattened curve kinetics.

Response:

Thank you for this comment. We made it clearer in the text.

11. "Besides, we can observe a decrease in the percentage of the population infected by the virus" A direct link between a flatter curve, a greater proportion of Removed individuals and total population infected is not sufficiently made clear.

Response:

Thank you for this comment. We made it clearer in the text.

12. "and the disease lasts for a shorter period" - from my understanding decreasing exposures per day prolongs the epidemic overall while slowing down the incidence rate.

Response:

Thank you for this comment. Yes, is the correct way to understand the figure. We made it clearer I the text.

13. "a SIR model was calibrated": "calibration" is usually understood as the preparation of

a model or device for accurate measurements by adjusting it to known standards. In this case, “applied”, “simulated” or “fitted” would be suitable to use.

Response:

Done, thank you for the comment.

14. Line 190: “contact-matrix (between susceptible)” – upon introducing a new term, provide more detailed information about it.

Response:

Done, thank you for the comment.

15. Footnote number 10 should be incorporated in the methods section.

Response:

Done, thank you for the comment.

Minor spelling, grammar and punctuation issues:

1. Line 9 : “could help flattens the epidemic curve”, remove the “s” in flattens.

Response:

Done, thank you for the comment.

2. Line 16: The WHO alerted, instead of “alleged”.

Response:

Done, thank you for the comment.

3. Line 21 : “there is a need to study”

Response:

Done, thank you for the comment.

4. Line 22: “Covid-19 diseases” is redundant, remove the word diseases.

Response:

Done, thank you for the comment.

5. Line 29 : “The government decided to publish daily reports”

Response:

Done, thank you for the comment.

6. Line 30 : "the number of deaths"

Response:

Done, thank you for the comment.

7. Line 31 : "These declarations ended on April 10, 2020, that which is why…". Remove "that".

Response:

Done, thank you for the comment.

8. Line 44 : Remove "while" in "suitable while when dealing"

Response:

Done, thank you for the comment.

9. Line 48: "Thus, the individual is Infectious until he is" - "Thus, the individual is infectious until they are…"; Also line 121.

Response:

Done, thank you for the comment.

10. Line 55 : "Supposed" instead of "Supposing a closed population…"

Response:

Done, thank you for the comment.

11. Line 59 : Substitute word infectious by infection in "During the infectious period "; Also line 80, 122.

Response:

Done, thank you for the comment.

12. Line 60 : "at a rate β"

Response:

Done, thank you for the comment.

13. Line 99 : "Marche 6". Remove "e"

Response:

Done, thank you for the comment.

14. Line 157: "to progressively reduce instead of to reduce progressively"

Response:

Done, thank you for the comment.

15. Figure 1: "recovery" should be replaced by "recovered" or "recoveries".

Response:

Done, thank you for the comment.

16. Figure 2: "total population"

Response:

Done, thank you for the comment.

17. Appendix: The "the" before letters denoting parameters should be removed ("distribution of the β").

Response:

Done, thank you for the comment.

Reviewer 2:

1. The citations are low and there are nearly daily publications on COVID-19 SIR models. Two are in NEJM and JAMA. The authors should cite these to let readers know this is standard.

Response:

Thank you for the comment. We added the JAMA article in our manuscript. We did not find the article on SIR models in NEJM. However, we found another article in Infectious Disease Modelling using SIR models.

2. The SIR model is the Kermack and McKendrick model not Kendrick "model known as a general stochastic epidemic model (Kendrick and Kermarck 1927; Jones 2007)".

Response:

Done, thank you for the comment.

3. Make a table of parameter meanings, description of how they are estimated and their units. The parameters all have standard names like effective contact rate etc. The authors should rely on these standards.

Response:

Thank you for the comment. We made the parameter meaning clearer in the text using their standard names.

4. The model doesn't include any time lag and relies on those who were tested. Were these symptomatic cases? If so, the authors should point out their model does not include asymptomatic cases that were infectious and thus represents an underestimate.

Response:

Thank for the comment. Actually, the tested individuals in our dataset were both symptomatic and asymptomatic cases.

5. The calculation of R0 can be done in different methods. The authors make an assumption which helps their calculation. They should cite Pauline van den Dreissche's work on calculating R0. Also, R0 may change based on controls and thus create a time-varying R0. This should be noted in the discussion. A constant R0 is based on the assumptions behind the model. If these assumptions change, then the R0 changes.

Response:

Thank you for the comment. We added this important point the last paragraph of our discussion/conclusion.

6. There is no data sharing plan or information.

Response:

Thank you for the comment. We wrote a data sharing plan and provided a link to access our dataset.

7. There are some places the language can be tightened up. The authors should review a few times to clean up the writing.

Response:

Thank you for the comment. we have used the services of a professional language editor.
