# Supplementary material for: Simulating the progression of the COVID-19 disease in Cameroon using SIR models
Source: PLoS One. 2020 Aug 25;15(8):e0237832. doi: 10.1371/journal.pone.0237832 (PMC7447022; doi:10.1371/journal.pone.0237832)
Supplement: Attachment — Submitted filename: Response to Reviewers.pdf [file pone.0237832.s002.pdf]

Review response letter

Dear Editor,

Thank you for giving us a new opportunity to revise our paper on "Simulating the progression of the COVID-19 disease in Cameroon using SIR models". The suggestions offered by the reviewers have been helpful and we also appreciate your insightful comments on revising some aspects of the paper.

We have included the reviewers' comments immediately after this letter and responded to them individually, indicating how we addressed each concern and describing the changes we have made.

Best regards,

Ulrich NGUEMDJO
Freeman MENO
Audric DONGFACK
Bruno VENTELOU

Reviewer #2:

1. There are still spelling errors for example on page 38, acute should be accurate.

Response:

Thank you for the comment, the paper has been sent for editing. It is now OK.

2. The time varying reproductive rate reference is not Pauline van den Dreissche's. Her article discusses ways to compute R0.

This article discusses time varying R0:
Massad E, Burattini MN, Lopez LF, Coutinho FA. Forecasting versus projection models in
epidemiology: the case of the SARS epidemics. Med Hypotheses. 2005;65(1):17-22. Epub 2005/05/17. doi: 10.1016/j.mehy.2004.09.029. PubMed PMID: 15893110; PubMed Central PMCID:
PMCPMC7116954.

Response:

Thank you for the comment. We added the reference in the manuscript.
